# Navigating Legitimacy in the Development of Obstructive Sleep Apnea Diagnostics: A Qualitative Study

**DOI:** 10.1177/00469580261448593

**Published:** 2026-05-03

**Authors:** Taru Kesävuori, Tero Montonen, Päivi Eriksson, Simcha Jong

**Affiliations:** 1 163043 Business School, University of Eastern Finland, Kuopio, Finland; 2 4919 Global Business School for Health, University College London, London, UK

**Keywords:** obstructive sleep apnea, diagnostic innovation, legitimacy navigation, legitimacy pressures, innovation adoption, digital health, health technology

## Abstract

**Introduction:**

Health technology innovations often challenge established practices, roles, and norms, which can impede their adoption and diffusion across healthcare systems. This study explores how those involved in sleep research and innovation facilitate adoption and diffusion of diagnostic solutions for obstructive sleep apnea (OSA) by addressing perceived legitimacy concerns—conceptualized here as cognitive, moral, and pragmatic legitimacy pressures—during solution development. We refer to these efforts as *navigating legitimacy*.

**Methods:**

The study draws on 24 thematic interviews with individuals involved in sleep research and innovation in Finland (e.g., researchers, diagnostic device developers, clinicians). Interview transcripts were analyzed using inductive and deductive thematic analysis.

**Results:**

The analysis indicated that respondents navigate legitimacy through contextual and ongoing legitimacy-building activities that combine discursive and material aspects. These activities exert influence on how the diagnostic solution takes shape, highlighting that diagnostic innovation is profoundly embedded within social and institutional contexts.

**Conclusion:**

Practically, our research provides valuable insights for researchers, developers, and policymakers into the dynamics through which digital health innovation is developed.

## 1. Introduction

In the field of health technologies, innovations often challenge established practices, roles, and norms, which can impede their adoption and complicate their diffusion across healthcare systems.^1^ For example, although telemedicine offers significant benefits, such as improved access to care and reduced costs, its adoption has remained limited due to resistance from healthcare professionals and the administrative and psychological challenges related to its implementation.^2^ Although various professionals—such as researchers, technology developers, and clinical actors—contribute to implementing these innovations, we know relatively little about how they work to facilitate the adoption and diffusion of such innovations during their development.

Drawing on institutional theory, this study explores this phenomenon by using the concept of *legitimacy*. In the literature, legitimacy is defined as a “generalized perception or assumption that the actions of an entity are desirable, proper, or appropriate within some socially constructed system of norms, values, beliefs and definitions”^3(p574)^. Legitimacy has attracted attention in the context of healthcare innovation, as studies have emphasized its importance for the implementation, diffusion, and integration of new treatments,^4^ practices,^5,6^ and technologies^1,7,8^ within healthcare systems.

Following Suchman’s^3^ seminal framework, researchers typically distinguish three main dimensions of innovation legitimacy: cognitive, moral, and pragmatic. *Cognitive legitimacy* refers to how comprehensible an innovation is within its social context.^9,10^ *Moral legitimacy* refers to an innovation’s alignment with the normative beliefs widely held in a given social context, such as whether the innovation abides by local laws, rules, and regulations.^9,10^ *Pragmatic legitimacy* is grounded in how well an innovation serves the practical needs of stakeholders.^9,10^ For example, Howe et al.^7^ examined the role of legitimacy in the integration of artificial intelligence (AI)-based technology into medical practice. Their work highlights how the successful implementation of health technology depends not only on technical functionality but also on how the technology gains acceptance from and/or is officially approved by healthcare institutions, such as regulatory bodies and insurance companies, and how it aligns with the working practices and expectations of healthcare professionals and users.

In this context, the literature explores how stakeholders interested in the diffusion of an innovation seek to enhance its legitimacy through activities often referred to as *legitimacy-building.* For example, Beaulieu and Lehoux^11^ show how health technology firms use political, associational, normative, and identity-related strategies during the procurement and distribution of medical devices to respond to expectations originating from financial and regulatory bodies, health professionals, and shareholders. Furthermore, in a recent article, Randhawa et al.^1^ explore how digital technology suppliers in the healthcare industry use the strategies of *solution selling* and *issue selling* to achieve legitimacy for their digital solutions among customer organizations and relevant field-level stakeholders. Solution selling refers to convincing the stakeholders of the concrete benefits of the technology, whereas issue selling focuses on highlighting the benefits of the change that the technology enables.^1^

Although the literature emphasizes the importance of legitimacy-building for the adoption and diffusion of innovations in healthcare systems, prior research has approached this topic from a relatively narrow perspective. For example, much of the existing empirical research views legitimacy-building as a post-development activity, despite evidence of its importance also in the early stages of innovation.^9,12^ Furthermore, research has highlighted the situated nature of legitimacy-building, showing that such activities are not universal but are shaped, for example, by innovation characteristics,^10,13^ context,^14^ and stakeholder expectations.^10^ For example, a recent study examining the development of digital health platforms (“netdoctors”) in Sweden demonstrated how the platforms’ business models were adapted in response to the expectations and reactions of ecosystem actors.^15^ However, despite growing interest, the situated nature of legitimacy-building has received relatively little attention in the field of digital health innovation.

To address these gaps, this study explores how those involved in sleep research and innovation in Finland build legitimacy when developing new digitally enabled diagnostic solutions for obstructive sleep apnea (OSA), a highly prevalent chronic sleep disorder characterized by repeated upper airway collapse during sleep.^16^ Specifically, we examine how these actors work to facilitate the adoption and diffusion of diagnostic solutions by addressing the perceived legitimacy concerns—conceptualized here as cognitive, moral, and pragmatic legitimacy pressures—during the solution development. We refer to these efforts as *navigating legitimacy*. With this concept, we distinguish such efforts from, on the one hand, legitimacy building activities that are reactive responses to pressures from the environment, and on the other hand, strategies that pay little attention to the context in which the diagnostic solutions are being developed.

OSA provides an interesting context for our study. The prevalence of OSA is increasing globally, with an estimated 936 million adults aged 30-69 years affected worldwide.^17^ In Finland, it was estimated that 1.46 million people (or 27% of the entire population) suffer from mild to severe sleep apnea.^17-19^ In recent years, alongside the digitalization of OSA management and initiatives to improve care efficiency and effectiveness,^20^ the diagnosis of OSA has evolved towards home-based, technology-driven, and telemedicine-enabled models.^21,22^ A key component of this shift has been the effort to provide an alternative to the gold standard of laboratory-based polysomnography (PSG), a diagnostic method whose limitations—such as high cost, time-consuming nature, and intensive resource requirements—are well documented in the literature.^23,24^ However, the widespread adoption of new OSA diagnostic solutions remains challenging.^22,24^ The solutions have faced resistance for example due to insufficient diagnostic accuracy, lack of patients’ acceptance and trust, regulatory constraints, and the strong grounding of existing diagnostic standards.^22,25^

Against this background, we ask the following research question: *How do those involved in sleep research and innovation navigate legitimacy when developing new digitally enabled diagnostic solutions for obstructive sleep apnea (OSA)?* In our study, the development is subjected to a general examination, without reference to a specific diagnostic solution or innovation project. This perspective enables an exploration of the legitimacy pressures and associated legitimacy-building activities across the sleep research and innovation field.

## 2. Methods

### 2.1. Study Design

A qualitative research approach using thematic interviews and reflexive thematic analysis was employed in this study.^26,27^ The study follows the Consolidated Criteria for Reporting Qualitative Research (COREQ) guidelines^28^ to support explicit and comprehensive reporting (see Supplemental Appendix A).

### 2.2. Participants

The data consisted of 24 thematic interviews with stakeholders involved in sleep research and innovation in Finland. The participants represented various stakeholder groups, including physicians, researchers, technology developers, and representatives of professional and patient organizations. They came from public (n=14), private (n=7), and third sector (n=3) organizations. Half of the participants had a clinical background, and the other half had a background in science, business, or patient advocacy. Some of the participants worked in a hybrid role. Table 1 below presents the participant profiles and interview data.

**Table 1.** Participant Profiles and Interview Data. Source: Data Collected by the Authors

Interview	Role	Type of institution (primary)	Sector	Interview length (min)	Length of transcript (pages)
1	Researcher	University	Public	54	12
2	Researcher	University	Public	118	49
3	Researcher	University	Public	65	25
4	Researcher	Hospital	Public	83	34
5	Researcher	University	Public	107	36
6	Sleep study specialist	University	Public	87	36
7	Sleep study specialist	University	Public	88	38
8	Regulatory affairs specialist	National regulatory agency	Public	56	16
9	Clinician-researcher	Hospital	Public	57	19
10	Clinician-researcher	Hospital	Public	52	19
11	Clinician-researcher	Hospital	Public	59	19
12	Clinician-researcher	Hospital	Public	69	20
13	Clinician-researcher	Hospital	Public	66	20
14	Clinician-researcher	Hospital	Public	57	23
15	Clinician-researcher	Service company	Private	47	15
16	Director	Service company	Private	54	19
17	Director	Tech company	Private	56	23
18	Product manager	Tech company	Private	85	29
19	CEO	Tech company	Private	98	29
20	CEO	Tech company	Private	57	20
21	CEO	Service company	Private	51	20
22	Patient organization representative	NGO	Third	60	16
23	Patient organization representative	NGO	Third	59	17
24	Professional association representative	NGO	Third	56	17
​	​	​	​	1641	571

The study participants were identified through professional networks, relevant conferences, publications, and the media, as well as from recommendations from earlier interviewees. The participants were selected through purposive sampling. The inclusion criterion required the participants to have at least 2 years of practical experience in sleep-related research or innovation work in Finland. This includes participating in sleep research and/or innovation as a researcher or developer, or contributing through commercial, facilitative, or advocacy roles. Individuals with less than 2 years of experience were excluded. In addition, individuals were excluded if their connection to sleep research and innovation was solely theoretical or administrative.

The potential participants were approached via email by the first author. They were sent an informed consent form and a participant information sheet describing the study’s focus on sleep research and innovation in Finland. The participants were informed that the interviewer was a doctoral researcher and that this study was part of a doctoral research project. In total, 36 individuals were contacted, of whom 24 agreed to participate. The most common reason for declining was lack of time. As participation was voluntary, individuals with stronger views or experiences may have been more likely or willing to participate. To address this, we purposively recruited participants from different sectors, organizations, and stakeholder groups to ensure sample diversity. In line with reflexive thematic analysis, the adequacy of the sample was evaluated based on the relevance and richness of the data (information power) rather than on saturation as a fixed endpoint.^29,30^ Participants were not known to the researchers prior to the study.

### 2.3. Data Collection

The interviews were conducted via Microsoft Teams® by the first author (female) between February and June 2023 from her home office in Finland. No other parties were present besides the first author and the participant. The interviews were audio- and video-recorded with the participants’ consent. The thematic interview guide was drafted by the first author and then discussed and revised with the co-authors (see Supplemental Appendix B). The interview guide consisted of open-ended questions and prompts related to the following three themes: the Finnish sleep research and innovation environment, the role of digitalization in sleep research and innovation, and the future of sleep research and innovation. Open-ended questions were employed to provide the heterogeneous group of participants an opportunity to highlight issues that they considered essential, timely, or important. The thematic interview guide was neither formally validated nor pilot tested. This approach aligns with our use of reflexive thematic method, where the focus is on responsiveness to participants’ perspectives rather than on standardization of the interview instrument.^27^ While prepared themes guided the interviews, most of the questions arose in the interaction between interviewer and participant, with both parties contributing to the co-construction of the data. Short notes were taken during the interviews to acknowledge and reflect on potential assumptions. Each participant was interviewed once, and the transcripts were not returned to participants for comment or correction. The interview audio, lasting an average of 68 min, was transcribed verbatim and uploaded to Atlas.ti software for qualitative data analysis.

### 2.4. Data Analysis

The interview data were analyzed using a combination of inductive (data-driven) and deductive (theory-driven) reflexive thematic analysis approaches.^26^ The analysis began with the authors thoroughly familiarizing themselves with the transcripts of the 24 interviews. The transcripts were read multiple times to get an overall understanding of the content. We observed that, in the interviews, the respondents described the factors that they perceived as influencing the adoption and diffusion of new diagnostic solutions for OSA, as well as how these factors were (or should be) considered to ensure the success of such innovations. We decided to investigate this phenomenon further.

The first author coded the interview transcripts inductively using Atlas.ti software. Because the reflexive thematic approach conceptualizes analysis as inherently researcher-generated rather than an objective procedure, analytic rigor was ensured through close reading of the data and transparent documentation of interpretive decisions.^26^ The codes were also regularly discussed within the research team. Inductive analysis allowed us to identify and construct initial themes of participants’ experiences related to diagnostic innovation for OSA. We began referring to these themes as “pressures” and “activities”.

Next, to deepen our conceptual understanding of the phenomenon, we shifted towards a theory-driven analysis. In this orientation, the data remained the foundation for coding, but the research question and codes were informed by theoretical concepts that guided the interpretation.^26^ We engaged with relevant literature and identified legitimacy theory as a useful framework for structuring and interpreting the identified pressures and activities. Furthermore, we introduced the term ‘legitimacy navigation’, as this term, in our view, best captured the ways in which the interviewees engaged with and responded to the perceived pressures. We defined our research question as follows: *how do those involved in sleep research and innovation navigate legitimacy when developing new digitally enabled diagnostic solutions for obstructive sleep apnea (OSA)?*

After that, we defined a priori codes representing the three main dimensions of legitimacy that are frequently identified in the literature (cognitive, moral, and pragmatic). Using Atlas.ti software, we applied these codes systematically to the interview data while simultaneously continuing data-driven coding. This allowed us to build on legitimacy theory, while remaining open to new insights.^31^

**Figure 1.**
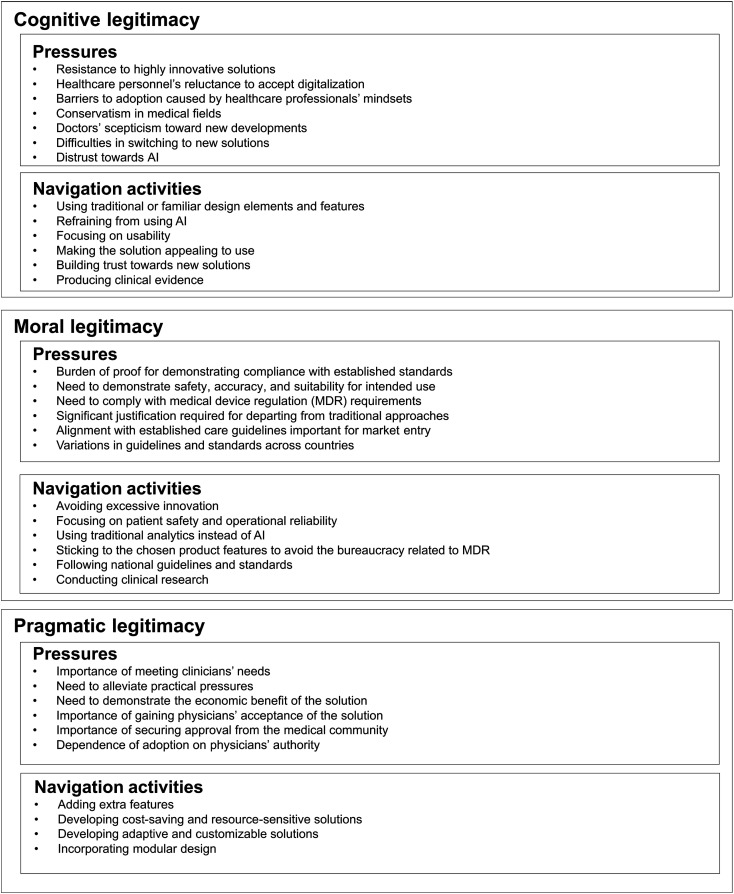
Themes established by thematic analysis of the 24 interviews. Source: Authors' analysis.

The analytical process was iterative, and it involved continuous transitions between reading, coding, interpretation, and synthesis. The codes and themes were reviewed and refined through discussions among the research team until consistency between the data and the findings was achieved. The research team consisted of a Doctoral Researcher in Innovation Management (MSc), an Assistant Professor of Innovation Management (PhD), a Professor of Innovation Management (PhD), and a Professor of Management and Healthcare Innovation (PhD). All team members had a background in organizational research, with some having stronger experience in healthcare contexts and others in innovation. These backgrounds influenced both the identification of the phenomenon and the interpretation of the data. Reflexivity was maintained through regular discussions within the research team. The first author documented these discussions in a research diary, enabling the team to revisit and reflect on analytical decisions and researchers’ positionalities. Finally, the themes were defined, examined in relation to one another, and named (Figure 1), forming the analytical framework presented and discussed in the Results section.

## 3. Results

This section reports the findings of our thematic analysis of the 24 interview transcripts. The findings are compiled in Table 2 and presented in more detail in the following sections.

**Table 2.** Navigating Legitimacy. Source: Authors’ Analysis

Navigating legitimacy in the development of obstructive sleep apnea diagnostics			
​	Cognitive legitimacy	Moral legitimacy	Pragmatic legitimacy
Pressure	Pressure to develop a diagnostic solution that is understandable, acceptable, and compatible with existing practices Background: Conservatism of the medical field	Pressure to develop a diagnostic solution that aligns with prevailing standards, recommendations, and criteria (e.g. MDR, national care guidelines, reimbursement criteria) Background: Burden of proof arising from the complex normative environment	Pressure to develop a diagnostic solution that meets the practical needs of key stakeholders (e.g., public sector entities, clinical personnel) Background: Critical role of key stakeholders in solution’s adoption and embedding
Navigation activities	• Aligning the solution with existing expectations and practices • Using familiar design elements and features to ease the cognitive barriers to change • Relying on traditional analytics instead of utilizing AI to ensure trust among physicians • Producing clinical evidence to validate product performance • Building trust in new solutions through publications, conferences, and stakeholder engagement	• Aligning the solutions with existing guidelines and standards of care • Adhering to established (e.g. already approved) product features to avoid the bureaucracy associated with the MDR process • Ensuring compliance with reimbursement criteria • Balancing innovation and tradition • Producing clinical evidence to influence prevailing understandings related to the current diagnostic approaches	• Aligning the solution design with the identified needs • Developing cost-saving and resource-sensitive solutions to help society with resource constraints • Developing customizable and modular solutions to enhance their suitability for different markets, needs, and individual preferences • Adding extra features to the solution to meet the needs of doctors and healthcare staff

### 3.1. Navigating Cognitive Legitimacy Pressure

The participants described a pressure to develop a diagnostic solution that is understandable, acceptable, and compatible with existing practices. This pressure stems from the perceived conservatism within the medical field, which hinders the adoption of new solutions. The participants highlighted healthcare professionals’ resistance to change, including scepticism toward new approaches and difficulty in changing already-established practices.

One must remember that all medical fields are very conservative. Most doctors, based on my experience, act in such a way that, when they were in school, what they learned there is the good thing, and everything that comes after that should be viewed with some suspicion …. Most doctors are the kind who just do their job. They think that if this system has always worked, then why should I switch to something else, even if it might be better. Switching to something new is always difficult. Most people see any change as a bad thing. Early adopters are always in the minority. (Interview 20, CEO, tech company)

The participants emphasized how AI has not yet gained cognitive acceptance in OSA diagnostics, pointing out the distrust expressed by healthcare professionals concerning the “black box” nature of AI systems.

Artificial intelligence methods only provide the result. They are black boxes that don’t reveal what they are analyzing from the signals. They simply tell you that “Okay, this person has severe sleep apnea”. The level of trust has not yet been achieved where they are fully believed in and accepted as actually working. (Interview 5, researcher, university)

The participants navigated cognitive legitimacy by aligning the new diagnostic solution with existing expectations and practices. For example, familiar design elements and features were used to ease the cognitive barriers to change.

Now that we are entering the sleep market, quite a few of our old customers have also become interested in offering this overnight polygraphy service because, in our solution, the platform is the same one they are already using, meaning they already know how to use it; they already have everything under control. (Interview 18, product manager, tech company)

Relying on traditional analytics instead of utilizing AI was seen to help ensure trust among physicians.

We perform traditional analytics precisely because there may be situations where AI doesn’t work—or even gives incorrect results. And if the doctor can’t access that analysis, or the intermediate steps, it can call into question the whole black box that the system operates in .... By doing it this way, we avoid issues related to trust. The reliability can then be verified by the doctor or sleep technician directly from the data file. (Interview 19, CEO, tech company)

Conducting clinical research to demonstrate aspects such as measurement accuracy, suitability, and safety was seen as an essential means of enhancing professionals’ confidence in the new solution. Publications, conferences, and stakeholder engagement were seen as important arenas to promote these viewpoints.

Conferences, webinars, seminars … present research findings and how they compare to current clinical practices, and through that, help to build trust in new methods. (Interview 5, researcher, university)

It requires close collaboration with clinical experts and the generation of clinical evidence. You have to do clinical studies to show ... of course, you always have to show that this solution is safe ... but also that it works. And it’s always better if the clinical study is done by an independent party. (Interview 20, CEO, tech company)

### 3.2. Navigating Moral Legitimacy Pressure

The participants described a pressure to develop a diagnostic solution that aligns with prevailing standards, recommendations, and criteria (e.g., Medical Device Regulation [MDR], national care guidelines, reimbursement criteria). This pressure arises from the perceived burden of proof, which pushes developers to adhere to established norms within the medical industry to ensure the solution’s adoption and diffusion in the healthcare system.

In the field of medical devices, there is a significant burden of proof—it’s necessary to demonstrate that the device is safe, accurate and suitable for its intended use. There are extremely lengthy standards that specify what must be met and how various aspects must be tested … It’s a massive undertaking for any company to meet these requirements and to provide the necessary evidence. (Interview 19, CEO, tech company)

When a solution doesn’t comply with the relevant clinical guidelines, entering the market becomes extremely difficult (Interview 20, CEO, tech company)

The participants described how MDR, as part of mandatory legislation, encourages operators to adhere to established product features to avoid the extensive bureaucracy—referred to as the “paper war”—associated with the MDR process.

The bureaucratic processes [related to MDR] are, of course, extremely lengthy. You have to quickly lock something in and start validating it. If you change it, you have to validate it all over again—and that takes several more years. The bureaucracy pushes you to choose one solution to commit to and fight the paper war with that. You can’t really keep developing the device or innovation continuously because each time you’d have to go through the whole bureaucratic process all over again. (Interview 4, researcher, hospital)

In addition to relevant laws, the perceived burden of proof also involves non-binding recommendations. For example, solutions that strictly adhere to national care guidelines and reimbursement criteria, even when it is not legally required, were perceived as having a competitive advantage in procurement processes.

A range of device options has emerged on the market, and it has been very interesting to observe their evolution. However, at the stage when these devices are being considered in competitive bidding processes, the key issue is whether they comply with national care guidelines or whether any deviations [from such guidelines] have been made in the devices. (Interview 21, CEO, service company)

No matter how good the product is or how well it works ... if it doesn’t meet the criteria for reimbursement set by each country—or by insurance providers in the U.S., for example—no one is going to buy it. (Interview 20, CEO, tech company)

The participants navigated moral legitimacy by balancing innovation and tradition. This was done by avoiding excessive novelty and ensuring that the diagnostic device complies with existing regulations, guidelines, and operational requirements.

We are a technology company, more of a high-tech business, so naturally we have all kinds of visions and ideas. But when it comes to medical devices—with all the regulations and heavy burden of proof—our strategy was to enter the market using very traditional methods. We didn’t go for some wild innovation project precisely because of that heavy proof requirement and all the related demands. So we’ve had to focus on the basics, really … patient safety, device reliability, and ease of use. (Interview 18, product manager, tech company)

Reimbursement criteria encouraged the addition of supplementary functionalities to the solution to facilitate its adoption and uptake in the market.

The goal is no longer solely to implement the best possible analytics but rather to begin by asking what is worth measuring in order to achieve the highest possible reimbursement levels… It is beneficial for us to develop an algorithm that measures sleep time, even though it doesn’t have that much significance in Finland …. It is beneficial for us globally …. It helps us enter markets more effectively in the U.S. (Interview 18, product manager, tech company)

The participants also sought to influence prevailing understandings related to the current diagnostic approaches by producing clinical evidence in support of their own solutions.

This is the way sleep apnea has always been measured, and the clinical guidelines state that this is what must be measured. We can argue that a much more reliable picture can be obtained by listening to breathing sounds from the throat ... but that’s not the traditional method. We have to prove that it is at least as reliable as, and preferably better than, the conventional approach. This requires clinical research. (Interview 20, CEO, tech company)

### 3.3. Navigating Pragmatic Legitimacy Pressure

The participants described a pressure to ensure that the diagnostic solution meets the practical needs of the key stakeholders (e.g., public sector entities, clinical personnel). The pressure arises from their perceived critical role in the solution’s adoption and embedding.

The participants highlighted the role of the public sector in diagnostic innovation. They described how the increasing number of OSA patients in Finland has challenged the capacity of public resources, creating a need for cost-effective solutions. For example, public healthcare is perceived as lacking the financial capacity to conduct extensive sleep studies.

If the number of sleep apnea patients increases dramatically—as is already happening, and is expected to continue growing exponentially for years—can our healthcare system afford to require that every person suspected of having sleep apnea undergo an EEG [Electroencephalography] before a diagnosis can be made? The answer is no. (Interview 12, clinician-researcher, hospital)

In a way, what’s most important when designing new software, for example, is being able to show that it might save money or that it’s at least the same price as the previous product while providing a health benefit. (Interview 9, clinician-researcher, hospital)

Another key stakeholder is clinical personnel—particularly physicians—whose influence is considered crucial for the adoption of new healthcare technologies. Meeting their practical needs was regarded as essential for the success of the solution.

[Doctors play a very important role in product development], since the solution is ultimately intended for their use. So the device should help relieve some of their pressure—whether it’s financial pressure or something else, like reducing wait times or improving resource efficiency. It’s important that they accept the solution in order for it to have a chance to succeed. (Interview 19, CEO, tech company)

The participants navigated pragmatic legitimacy by aligning the solution design with the identified needs. This had led to growing interest in home diagnostic solutions, as they simplify logistics and place less burden on the healthcare system compared with traditional laboratory-based sleep studies.

Since laboratory measurements are quite expensive and labour-intensive, an alternative would be developed—one that is reliable and allows everything to be done easily at home. This naturally makes things easier for everyone: the diagnosing physician and the healthcare system are not burdened as much. (Interview 6, sleep study specialist, university)

To meet the needs of healthcare personnel, the user interface of the solutions was designed to be customizable, enabling doctors and other healthcare staff to use it easily. Modular solutions were seen to provide flexibility for the users to tailor the system to their needs and preferences.

There is a great deal of interest in the analytics software—particularly in the quality of the signals it produces. Key considerations include how the physician views different biosignals, how they are visualized in the analytics software, what kind of algorithms are used, how effectively they detect relevant events and how well the software pre-analyses those events. These aspects are highly valued. Additionally, the user interface plays an important role: how simple it is to use, whether it is adaptive and whether I, as a physician, can customize it to match the way I’m used to working. The physician’s work often becomes a bottleneck in the entire process, so making their work easier or faster has been a critical factor. (Interview 18, product manager, tech company)

Furthermore, additional features that were considered useful for key stakeholders were incorporated into the solution. For instance, an electrocardiogram (ECG) function was added because it was believed that doctors would find it valuable.

We included ECG as one of the parameters .... It may not be strictly necessary, but we wanted to incorporate it—many doctors are keen to see whether arrhythmias occur during sleep-related breathing disorder events. (Interview 18, product manager, tech company)

## 4. Discussion

Drawing on 24 thematic interviews, this study examined how stakeholders in the field of sleep research and innovation in Finland work to facilitate the adoption and diffusion of diagnostic solutions for OSA by addressing the perceived legitimacy concerns during the solution development.

This study provides the following main contributions. First, rather than being something that happens only after an innovation has been developed, in our study legitimacy-building emerges as an ongoing activity integral to shaping innovations from an early stage. This aligns with Blume’s^32^ argument that innovation diffusion cannot be clearly separated from preceding and concurrent development work, suggesting that the processes of developing and legitimizing innovation evolve simultaneously.

Second, by introducing the concept of *legitimacy navigation*, our findings portray legitimacy-building as a contextually situated activity in which the respondents in our study creatively shape the development work and the diagnostic solutions to the pressures they perceive. These findings align with previous research emphasizing the significance of contextual factors in legitimacy-building^10,13,14^ and healthcare innovation.^33,34^

Our third contribution lies in highlighting the material aspects of processes for establishing legitimacy in digital health innovation. Previous research has primarily emphasized discursive practices portraying legitimacy-building as involving rhetorical and communicative activity that relies on language, framing and discourse as key resources for legitimation.^35-37^ However, material aspects of legitimation have remained largely unexplored, leaving our understanding of these processes incomplete.^36^ Our study addresses this limitation by showing how the respondents navigated legitimacy pressures not only through discursive strategies—such as shaping perceptions through publications, conferences and stakeholder engagement—but also by materially aligning, adapting, configuring, and tailoring the diagnostic solution to meet contextual and institutional expectations. These findings advance the understanding of “how matter matters” ^36(p3)^ in legitimation practices and highlight materiality as an active component of legitimacy-building in digital health innovation.

**Figure 2.**
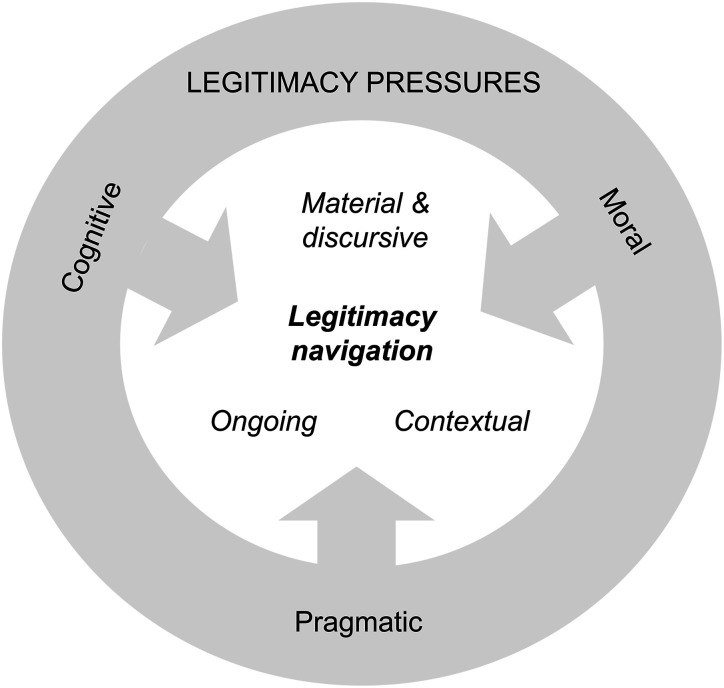
Legitimacy navigation. Source: Authors’ conceptual model developed from study findings.

Practically, our research is valuable for researchers, developers, and those who shape policies and practices in the healthcare sector by providing insights into the dynamics through which digital health innovation is developed (Figure 2). For example, in our empirical analysis, we showed how perceived legitimacy pressures were navigated through activities aimed at enhancing the likelihood that an innovation is adopted, sometimes at the expense of novelty. By encouraging familiar, easily adoptable solutions and adherence to established guidelines and recommendations even when not legally required, these pressures may reduce the likelihood that technological breakthroughs will be introduced. As government budget allocations for research and development in Finland have increased since the mid-2010s,^38^ it has become particularly important to understand how contextual factors shape healthcare innovation.^34,39^

The data used in this study were collected from interviews with individuals operating primarily within Finland, a Nordic welfare state in which healthcare is predominantly publicly funded. In 2021, around 80% of current health expenditure was financed from public sources in Finland.^40^ In this context, the emphasis on pragmatic legitimacy—especially the pressure to meet the public sector expectations by developing cost-effective diagnostic solutions—becomes understandable. This was especially evident in the interviews with participants closer to everyday clinical practice (e.g., clinician-researchers, sleep study specialists). In addition, the interviews were conducted at a time when regulatory requirements for medical devices had tightened following the introduction of the European Union’s Medical Device Regulation (MDR).^41^ This shift in regulatory environment may have increased the salience of moral pressures, particularly in the interviews with participants representing technology companies. Furthermore, the study focuses on a single condition, OSA, which has its own specific characteristics reflected in diagnostic practices. Concentrating on one particular disorder in one country this way limits the generalizability of the findings internationally or to other diagnostic settings. While the data should be interpreted with this specific context in mind, these limitations also highlight the need for further exploration. Future research could examine legitimacy-building across different healthcare systems and diagnostic contexts to assess whether the patterns observed here reflect broader trends in digital health innovation.

## 5. Conclusion

This study sheds light on how individuals involved in sleep research and innovation build legitimacy when developing new diagnostic solutions for OSA in Finland. Based on our analysis of 24 interviews, we demonstrated how these actors work to facilitate the adoption and diffusion of diagnostic solutions through legitimacy-building activities that are contextual and ongoing, including both material and discursive aspects. Through these activities, the actors address perceived legitimacy concerns related to the solution’s understandability, acceptability, and compatibility with existing practices (cognitive legitimacy); its alignment with prevailing standards, recommendations, and criteria (moral legitimacy); and its ability to meet stakeholders’ practical needs (pragmatic legitimacy). These activities—referred to here as *legitimacy navigation*—influence how the diagnostic solution takes shape, underlining that digital health innovation is deeply embedded within social and institutional contexts.

## Supplemental Material

Supplemental Material - Navigating Legitimacy in the Development of Obstructive Sleep Apnea Diagnostics: A Qualitative Study Supplemental Material for Navigating Legitimacy in the Development of Obstructive Sleep Apnea Diagnostics: A Qualitative Study by Taru Kesävuori, Tero Montonen, Päivi Eriksson and Simcha Jong in Inquiry: The Journal of Health Care Organization, Provision, and Financing.

Supplemental Material - Navigating Legitimacy in the Development of Obstructive Sleep Apnea Diagnostics: A Qualitative Study Supplemental Material for Navigating Legitimacy in the Development of Obstructive Sleep Apnea Diagnostics: A Qualitative Study by Taru Kesävuori, Tero Montonen, Päivi Eriksson and Simcha Jong in Inquiry: The Journal of Health Care Organization, Provision, and Financing.
