# Supplementary material for: Navigating Legitimacy in the Development of Obstructive Sleep Apnea Diagnostics: A Qualitative Study
Source: Inquiry. 2026 May 3;63:1. doi: 10.1177/00469580261448593 (PMC13158497; doi:10.1177/00469580261448593)
Supplement: Supplemental Material - Navigating Legitimacy in the Development of Obstructive Sleep Apnea Diagnostics: A Qualitative Study [file sj-pdf-2-inq-10.1177_00469580261448593.pdf]

Thematic interview guide		
Introductions; confirming informed consent and confidentiality		
**Themes**	**Key questions**	**Prompts (examples)**
1. Finnish sleep research and innovation environment	*How would you describe the current field of sleep research and innovation in Finland?*	• *Could you share some practical experiences from sleep research and/or innovation work in Finland?*<br>• *Who do you see as the important stakeholders in this field? Why?*
2. Role of digitalization in sleep research and innovation	*How would you describe the role of digitalization in sleep research and innovation?*	• *How has digitalization appeared in your own work (if at all)?*<br>• *When you think about digitalization in this field, what kinds of possibilities or challenges (if any) come to your mind? Could you share any examples?*
3. Future of sleep research and innovation	*How would you describe the future of sleep research and innovation?*	• *What kinds of developments or trends have you noticed in recent years (if any)?*<br>• *When you think about the future, are there areas that you feel might benefit from change of further development? If so, in what ways?*
Concluding remarks; thanking the participant, opportunity for final comments or questions, explaining next steps		
